# Comparison of ultraconserved elements (UCEs) to microsatellite markers for the study of avian hybrid zones: a test in *Aphelocoma* jays

**DOI:** 10.1186/s13104-019-4481-z

**Published:** 2019-07-24

**Authors:** Nicholas T. Vinciguerra, Whitney L. E. Tsai, Brant C. Faircloth, John E. McCormack

**Affiliations:** 1 Moore Laboratory of Zoology, Occidental College, Los Angeles, CA USA; 2 Department of Biological Sciences, Louisiana State University, Baton Rouge, LA USA; 3 Museum of Natural Science, Louisiana State University, Baton Rouge, LA USA; 4 Biology Department, Occidental College, Los Angeles, CA USA; 5 Present Address: Department of Biology, San Diego State University, San Diego, USA

**Keywords:** Hybrid zones, Sequence capture, Population genomics, Ecomorphology

## Abstract

**Objective:**

Hybrid zones are geographic regions where genetically distinct taxa interbreed, resulting in offspring of mixed ancestry. California Scrub-Jays (*Aphelocoma californica*) and Woodhouse’s Scrub-Jays (*A. woodhouseii*) come into secondary contact and hybridize in western Nevada. Although previous work investigated divergence and gene flow between these species using a handful of microsatellite markers, the hybrid zone has not been studied using genome-scale markers, providing an opportunity to assess genome-wide introgression, test for a genetic basis for ecomorphological traits, and compare these estimates to those derived from microsatellites.

**Results:**

Using variant sites flanking ultraconserved elements (UCEs), we performed population assignment and quantified hybrid ancestry for 16 individuals across the zone of secondary contact. Our study included 2468 SNPs distributed throughout the genome, allowing discrimination of genetic affinities of hybrid individuals that were similar to estimates from microsatellites. We show a relationship between bill and wing length and the genetic composition of individuals that was not found in prior work using microsatellites, suggesting a genetic basis for these traits. Our analyses demonstrate the utility of UCEs for the analysis of hybrid zones and provide a basis for future studies to identify the genomic architecture of speciation and phenotypic differences between these incipient species.

**Electronic supplementary material:**

The online version of this article (10.1186/s13104-019-4481-z) contains supplementary material, which is available to authorized users.

## Introduction

Hybrid zones are windows into the process of species formation [1, 2]. The standard model of speciation starts when the geographic range of an ancestral species is fragmented, and over time, phenotypes and genomes diverge through processes that change allele frequencies [3–5]. In some cases, ranges may shift, allowing divergent lineages to come back into secondary contact. These zones of secondary contact provide an opportunity to analyze the evolution of species barriers and trait variation [6] as well as to examine the genomic architecture of phenotypic differences [7, 8].

Molecular marker development is changing the way hybrid zones are studied, allowing genome scale inferences. Whole genomes are becoming the gold standard for hybrid zone studies [9], but whole-genome data are still relatively expensive to collect and computationally demanding to analyze. Restriction site-associated DNA sequencing (RADSeq) data provide a more cost-effective mechanism to collect genome-scale data and have been used extensively in hybrid zone work, but standard RADseq approaches do not work well with some types of DNA, especially degraded DNA from museum specimens. Ultraconserved elements (UCEs) are one type of molecular marker that have proved useful for collecting genomic-level phylogenetic data at both deep and shallow timescales [10, 11]. UCEs have not often been used to study hybrid zones [but see 12, 13], although they are a potentially useful way to collect non-coding DNA, similar to introns or microsatellites, at the genome scale. However, the target enrichment method for collecting UCEs—where the conserved elements act as a central anchor with variation primarily confined to the flanking regions—could make it difficult to assemble enough single nucleotide polymorphisms (SNPs) from UCEs to effectively study the recent divergences involved in most hybrid zones. Also, it is unclear to what extent UCE results may differ from population genetic markers like microsatellites because of the different mutational model that may underlie the evolution of UCE regions [14].

Here, we compare two molecular marker datasets across an avian hybrid zone to test if SNPs mined from UCEs give similar results to prior microsatellite work, and we use the genome-scale UCE data to assess whether some ecomorphological traits have a genetic basis. Previously, Gowen et al. [15] examined patterns of genetic variation across the distribution of California Scrub-Jay (*Aphelocoma californica*) and Woodhouse’s Scrub-Jay (*A. woodhouseii*) using 13 microsatellite loci. These two recently-split species [16] show differences in bill shape: *A. californica* has a more heavy, hooked bill compared to the narrower and longer bill of *A. woodhouseii*; these differences are thought to be adaptations to local resources [17, 18]. However, Gowen et al. [15] examined individuals of varying hybrid ancestry in the middle of the hybrid zone, where effects of the environment were minimized, and found that plumage traits were associated with genetic profile but bill traits were not. Failure to detect a correlation between genetic profile and bill divergence could have resulted from variance in the estimates of hybrid ancestry, because they were estimated from a small number of loci or because of stabilizing selection or plasticity in bill traits.

To determine if UCE data could shed light on this question, we first assessed whether sequence capture of UCEs would collect enough informative SNPs to determine genetic affinities of individuals. Then, we compared individual-level population assignment based on UCE data to that estimated from a previous dataset of 13 microsatellites for the same individuals [15]. Finally, we used our estimates of hybrid ancestry to test whether neutral genetic assignment could predict a suite of morphological traits, including those with a prior ecomorphological hypothesis. A correlation between hybrid ancestry and phenotypic traits in the middle of the hybrid zone would suggest these traits have an underlying genetic basis.

## Main text

### Methods

**Fig. 1:**
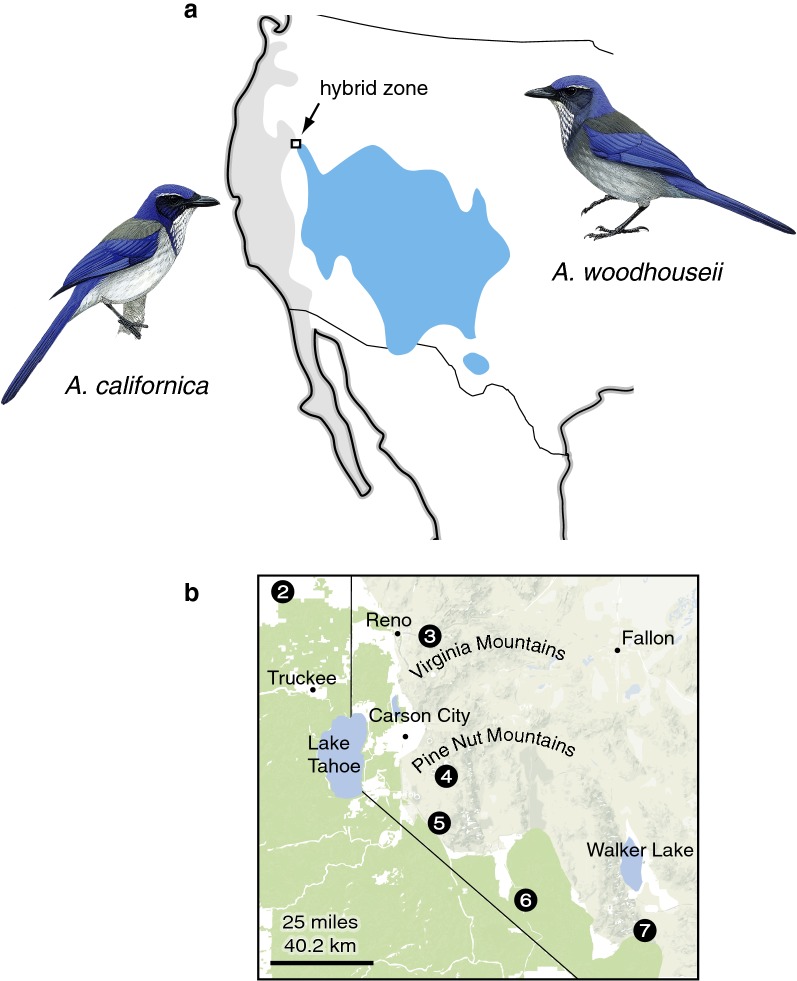
Species range map and location of the *Aphelocoma* jay hybrid zone. **a** Range of *A. californica* (gray) and *A. woodhouseii* (blue). **b** Inset of sampling sites in the hybrid zone (sites 1 and 8 on the ends of the hybrid cline are not depicted in the inset). Map modified from McCormack et al. [29] with permission and bird illustrations reproduced by permission of Lynx Edicions as they appear in Handbook of the Birds of the World Alive [30]

We obtained tissues from 16 vouchered museum specimens representing two individuals from each of eight populations of *Aphelocoma californica*, *A. woodhouseii*, and hybrids along a 722 km transect running northwest to southeast from the Sierra Nevada in northeastern California into west-central Nevada (Fig. 1; Table 1). This line corresponds to prior descriptions of the secondary contact zone [15, 19]. Methods for DNA extraction, library preparation, sequence capture, and variant calling are provided in Additional file 1.

**Table 1:** Museum specimens collected along transect through hybrid zone and estimates of hybrid ancestry (*Q*)

Museum^a^	Specimen no.	Collection date	Site^b^	Location	Latitude	Longitude	Distance (km)^c^	*Q*_SNPs_	*Q*_msats_
FMNH	333928	9 Jun. 1987	1	Alturas, CA	41.55	− 120.66	0	0.97	0.91
FMNH	333930	9 Jun. 1987	1	Alturas, CA	41.55	− 120.66	0	1.00	0.94
FMNH	333949	11 Jun. 1987	2	Beckwourth, CA	39.75	− 120.37	202	0.99	0.97
FMNH	333950	11 Jun. 1987	2	Beckwourth, CA	39.75	− 120.37	202	0.99	0.95
FMNH	333991	15 Jun. 1987	3	Virginia Mts, NV	39.50	− 119.63	279	0.76	0.67
FMNH	333994	15 Jun. 1987	3	Virginia Mts, NV	39.50	− 119.63	279	0.76	0.07
MVZ	179861	29 Sept. 2001	4	Pine Nut Mts, NV	39.06	− 119.57	292	0.44	0.63
MVZ	179865	29 Sept. 2001	4	Pine Nut Mts, NV	39.06	− 119.57	292	0.52	0.61
FMNH	333999	27 Jun. 1987	5	Gardnerville, NV	38.83	− 119.62	314	0.52	0.90
FMNH	334000	27 Jun. 1987	5	Gardnerville, NV	38.83	− 119.62	314	0.55	0.77
MVZ	178496	9 Dec. 1998	6	Nye Canyon, NV	38.57	− 119.21	353	0.02	0.03
MVZ	178497	9 Dec. 1998	6	Nye Canyon, NV	38.57	− 119.21	353	0.06	0.07
MVZ	179171	7 Dec. 1999	7	Wassuk Range, NV	38.46	− 118.65	384	0.03	0.04
MVZ	179172	7 Dec. 1999	7	Wassuk Range, NV	38.46	− 118.65	384	0.11	0.06
FMNH	334086	30 Mar. 1987	8	Mt. Charleston, NV	36.37	− 115.63	722	0.02	0.28
FMNH	334087	30 Mar. 1987	8	Mt. Charleston, NV	36.37	− 115.63	722	0.01	0.02

^a^FMNH, Field Museum of Natural History (Chicago); MVZ, Museum of Vertebrate Zoology (Berkeley)

^b^Sites 2–6 correspond to sampling localities in Fig. 1b. Specimens from sites 1 and 8 were collected outside the area of contact and hybridization

^c^Distance (km) represents Euclidean distance across the hybrid zone

To assess population assignment and obtain estimates of individual-level hybrid ancestry (*Q* scores), we analyzed the SNP dataset in STRUCTURE v2.3.4 [20] using *K* = 2. The *Q* score reflects the estimated proportion of nuclear variation inherited from *A. californica* or *A. woodhouseii*. We ran three independent analyses for each value of *K* = 2, using the admixture model and correlated allele frequencies. Each run had a burn-in of 100,000 generations and 1,000,000 Markov chain Monte Carlo generations post burn-in. We calculated per-locus *F*_ST_ on SNPs within the core UCE region, regions flanking UCEs, and microsatellites from Gowen et al. [15, 21] in the R package HIERFSTAT v0.04-22 [22] along with 1000 bootstrap replicates.

We conducted linear regression analyses of morphological traits with estimates of *Q* scores from STRUCTURE for individuals within the hybrid zone, to minimize effects of environmental differences and determine the extent to which nuclear DNA variation could predict phenotypic differences. The morphological dataset included measurements of bill width, bill length, bill depth, wing length, tail length, and tarsus length [15, 21]. Morphological characters were analyzed with principal components analysis in R v3.4.0 (http://www.r-project.org/) to identify axes defining the most variation. We assessed relationships between *Q* scores and univariate and multivariate morphological traits at two spatial scales: across the oak woodland and pinyon-juniper woodland (hybrid cline, where there are environmental differences between ends of the cline) and within the immediate area of contact (where all individuals are experiencing the same habitat and the effect of environment as a source of variation is minimized).

### Results

We collected ca. 1.9 × 10^8^ raw reads from each Illumina library. We retained 99% of the read data after trimming of low-quality bases and removal of adapter contamination (see Additional file 2 for DNA sequencing statistics). After quality control, we assembled cleaned reads from specimen FMNH 333991 into consensus contigs and filtered to identify UCE loci, resulting in 4308 reference UCE sequences with an average length of 737 bp. Between 20 and 25% of cleaned reads were mapped to the reference for SNP calling. The rest of the reads were off-target sequence. An average of 10% of reads per individual were identified and removed as polymerase chain reaction duplicates. After SNP calling and variant filtration, our SNP dataset included 2468 SNPs, with one SNP out of every 500 bp, or 1–2 SNPs per UCE locus on average. Each SNP had an average depth of coverage of 69.73 per locus (SD: 39.66).

**Fig. 2:**
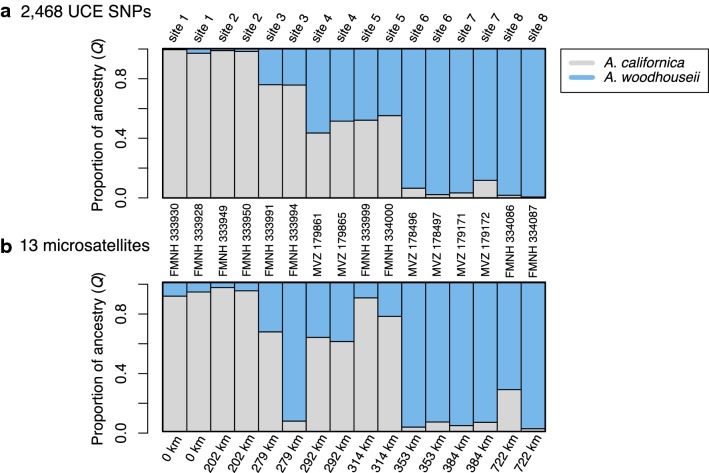
STRUCTURE results (*K *= 2). STRUCTURE analysis based on (**a**) 2468 UCE SNPs, (**b**) 13 microsatellites [15, 21]. The y-axis indicates assignment probability to either the *Aphelocoma californica* genetic cluster (gray) or the *A. woodhouseii* genetic cluster (blue). Each colored vertical bar represents a vouchered museum specimen and numbers along the x-axis indicate distance (km) along the sampling transect

STRUCTURE analysis of SNP data revealed a distinct geographical pattern of genomic variation across the hybrid zone (Fig. 2a). The 722 km transect running from northeastern California into west-central Nevada showed a clear relationship between geographic locality and relative assignment probability to each species. Individuals from ends of the transect were assigned to either *A. californica* or *A. woodhouseii* with ~ 99% probability, whereas individuals sampled from localities in between were assigned different proportions of hybrid ancestry.

Microsatellite-based *Q* scores from prior research were significantly correlated with SNP-based *Q* scores and explained ~ 69% of the total variation in SNP-based estimates (*R*-squared = 0.688; *p* = < 0.0005; Additional file 3: Figure S1). However, a few individuals’ genetic profiles differed between microsatellite and SNP data, and the qualitative pattern of genomic change across the hybrid zone was smoother with SNP data (Fig. 2).

Estimates of *F*_ST_ from SNPs flanking UCEs were greater than estimates from core UCE SNPs, as expected (flanking SNPs: average per-locus *F*_ST_ = 0.108, SD = 0.008; core SNPs: average per-locus *F*_ST_ = 0.073, SD = 0.013; Additional file 4: Figure S2). Microsatellite-based *F*_ST_ estimates were intermediate between these values, with larger variance likely due to smaller locus sample size (average per-locus *F*_ST_ = 0.129, SD = 0.042).

Although sample sizes were small, there were significant correlations between estimates of hybrid ancestry and two ecomorphological traits: wing length and bill length (Additional file 5: Table S2). For the dataset of 12 hybrid individuals (i.e., excluding the ends of the transect), wing length explained ~ 52% of the variation in *Q* score (*R*-squared = 0.524; *p* = 0.008). Narrowing the focus to the area of contact, where environmental differences are minimized, *Q* score explained ~ 67% of the variation in bill length (*R*-squared = 0.669; *p* = 0.047).

### Discussion

These results add to previous findings demonstrating that UCEs are informative genomic markers for addressing questions at shallow evolutionary timescales [11, 23–26] such as the divergences observed in hybrid zone studies [12, 13]. We show that SNPs mined from UCEs provide sufficient variation to infer patterns of divergence between incipient species in secondary contact with ongoing gene flow. Specifically, we use genome-scale data to confirm a smooth transition in genetic ancestry from *A. californica* to *A. woodhouseii* across a geographic cline, with individuals in the two middle sites (i.e. 4, 5) having nearly 50/50 ancestry estimated using our SNP data.

We also show that hybrid ancestry estimated from ~ 2400 SNPs drawn from UCEs are correlated with estimates from the 13 microsatellites, especially for individuals predicted to be of pure or nearly pure ancestry of one species (i.e. those at the ends of the cline). Individuals in the 4 middle sites (i.e. sites 3–6) showed less correlation between SNP-based and microsatellite-based *Q* scores than individuals at the 4 end sites (average difference in middle sites = 0.21; average difference of end sites = 0.07), although sample sizes were low and this effect was not significant (*p* = 0.11). While it is difficult to know which *Q* score is the more accurate measure because we do not know the true ancestry of individuals, hybrid values from SNP data were more similar for the two individuals within sites (average difference between individuals within sites for SNPs = 0.03; and for microsatellites = 0.14, *p* = 0.17), suggesting SNP data might be, if not more accurate, at least more precise than the microsatellite data.

More precise estimates of hybrid ancestry might help determine whether morphological traits have a genetic basis. Building off prior work on these species [15, 17, 18], we reasoned that we could minimize the effect of environment as a cause of the phenotypic differences between species by restricting the analysis to the zone of secondary contact, where individuals with a variety of hybrid scores experience the same environment. If phenotypic differences were driven by genetic mechanisms, then an individual with a higher proportion of *A. californica* DNA would also be more likely to have a short and stout bill, provided bill shape is under genetic control. However, there is considerable room for noise in this kind of analysis, because bill size and shape are likely polygenic [27] and the randomness of recombination means that an individual with a high proportion of *A. californica* DNA might nevertheless have inherited *A. woodhouseii* genes that affect bill dimensions. Despite these caveats, and our low sample size, we found an association between hybrid score and both bill length and wing length. This finding sets the stage for follow-up studies with larger sample sizes where pure individuals and natural backcrosses could be analyzed using whole-genome analyses to study the genomic underpinnings of these traits.

Hybrids zones are natural laboratories for investigating speciation and the genomic architecture of phenotypic traits. Here, we show that UCEs, and SNPs contained within them, are promising markers for the study of hybrid zones. Although UCEs do not typically occur within protein-coding genes when collected from vertebrate taxa, SNPs within UCE loci are amenable for studying so-called “neutral” processes in a similar manner as they are used currently in phylogeography and phylogenetics. Moreover, UCEs are universal across major taxonomic groups, abundant throughout the genome, efficient to collect, and useful with available tissue types, including degraded DNA extracted from museum specimens [28]. For these reasons, UCEs provide a useful genomic marker for studies seeking to understand the nature of divergence and gene flow between lineages in secondary contact.

## Limitations

Whole genome sequences and more individuals would be needed to identify the genomic underpinnings of speciation and phenotypic differences between these species.

## Additional files

**Additional file 1.** Methods for DNA extraction, library preparation, sequence capture, and variant calling.

**Additional file 2: Table S1.** Statistics on sequenced DNA data for individual museum specimens.

**Additional file 3: Figure S1.** Linear regression of hybrid ancestry (*Q* scores) estimates.

**Additional file 4: Figure S2.** Bootstrapped average per-locus *F*_ST_ values.

**Additional file 5: Table S2.** *R*^2^ values between hybrid index and phenotypic traits.

**Additional file 6.** VCF file of SNPs from UCE loci.

## Data Availability

The SNP dataset analyzed in this study is available in Additional file 6. The microsatellite dataset and morphological dataset are available for download from Dryad, 10.5061/dryad.57f48 [21].

## Abbreviations

RADSeq: restriction site-associated DNA sequencing
UCE: ultraconserved element
SNP: single nucleotide polymorphism
SD: standard deviation
