# Supplementary material for: Comparison of ultraconserved elements (UCEs) to microsatellite markers for the study of avian hybrid zones: a test in Aphelocoma jays
Source: BMC Res Notes. 2019 Jul 24;12:456. doi: 10.1186/s13104-019-4481-z (PMC6657088; doi:10.1186/s13104-019-4481-z)
Supplement: Supplementary file 1 — Additional file 1. Methods for DNA extraction, library preparation, sequence capture, and variant calling. [file 13104_2019_4481_MOESM1_ESM.docx]

**Methods supplement**

We extracted whole genomic DNA from tissue samples using the Qiagen DNeasy tissue kit. We fragmented genomic DNA using a BioRuptor NGS (Diagenode) and prepared Illumina libraries using Kapa library preparation kits and custom TruSeq adapters unique to each sample [1]. We followed the protocol for library preparation and target enrichment of UCEs from Faircloth et al. [2]. Two pools of eight samples each were enriched for 5060 UCE loci [2] using a set of synthetic RNA probes (Mycroarray, Inc.). We qPCR-quantified enriched pools, combined pools at equimolar ratios, and sequenced the combined libraries using PE250 sequencing on one run of an Illumina MiSeq.

We used PHYLUCE v1.5 [3] to perform quality control on the demultiplexed reads, to assemble reads into contigs, and to identify assembled contigs as UCE loci. We chose the specimen with the greatest number of recovered UCE loci (FMNH 333991) as the reference assembly for single nucleotide polymorphism (SNP) calling. We indexed the reference using SAMTOOLS v0.1.19 [4] and used BWA v0.7.7 [5] for read mapping. We removed PCR duplicates from the resulting BAM files with PICARD (http://broadinstitute.github.io/picard) and used GATK v3.2 [6] to realign the mapped reads around indels, to call variants, to quality-filter, to remove indels, and to select only biallelic SNPs. We used VCFTOOLS v0.1.14 [7] to eliminate all sites with missing data and to retain SNPs with a genotype quality ≥ 30.

**References**

1. Faircloth BC, Glenn TC. Not all sequence tags are created equal: designing and validating sequence identification tags robust to indels. PloS one. 2012;7:e42543.
2. Faircloth BC, McCormack JE, Crawford NG, Harvey MG, Brumfield RT, Glenn TC. Ultraconserved elements anchor thousands of genetic markers spanning multiple evolutionary timescales. Syst Biol. 2012;61:717–726.
3. Faircloth BC. PHYLUCE is a software package for the analysis of conserved genomic loci. Bioinformatics. 2015;32:786–788.
4. Li H, Handsaker B, Wysoker A, Fennell T, Ruan J, Homer N, Marth G, Abecasis G, Durbin R. The sequence alignment/map format and SAMtools. Bioinformatics. 2009;25:2078–2079.
5. Li H, Durbin R. Fast and accurate short read alignment with Burrows–Wheeler transform. Bioinformatics. 2009;25:1754–1760.
6. McKenna A, Hanna M, Banks E, Sivachenko A, Cibulskis K, Kernytsky A, Garimella K, Altshuler D, Gabriel S, Daly M, DePristo MA. The Genome Analysis Toolkit: A MapReduce framework for analyzing next-generation DNA sequencing data. Genome Res. 2010;20:1297–1303.
7. Danecek P, Auton A, Abecasis G, Albers CA, Banks E, DePristo MA, Handsaker RE, Lunter G, Marth GT, Sherry ST, McVean G. The Variant Call Format and VCFtools. Bioinformatics. 2011;27:2156–2158.
