# Supplementary figures and images for: Comparison of ultraconserved elements (UCEs) to microsatellite markers for the study of avian hybrid zones: a test in Aphelocoma jays
Source: BMC Res Notes. 2019 Jul 24;12:456. doi: 10.1186/s13104-019-4481-z (PMC6657088; doi:10.1186/s13104-019-4481-z)

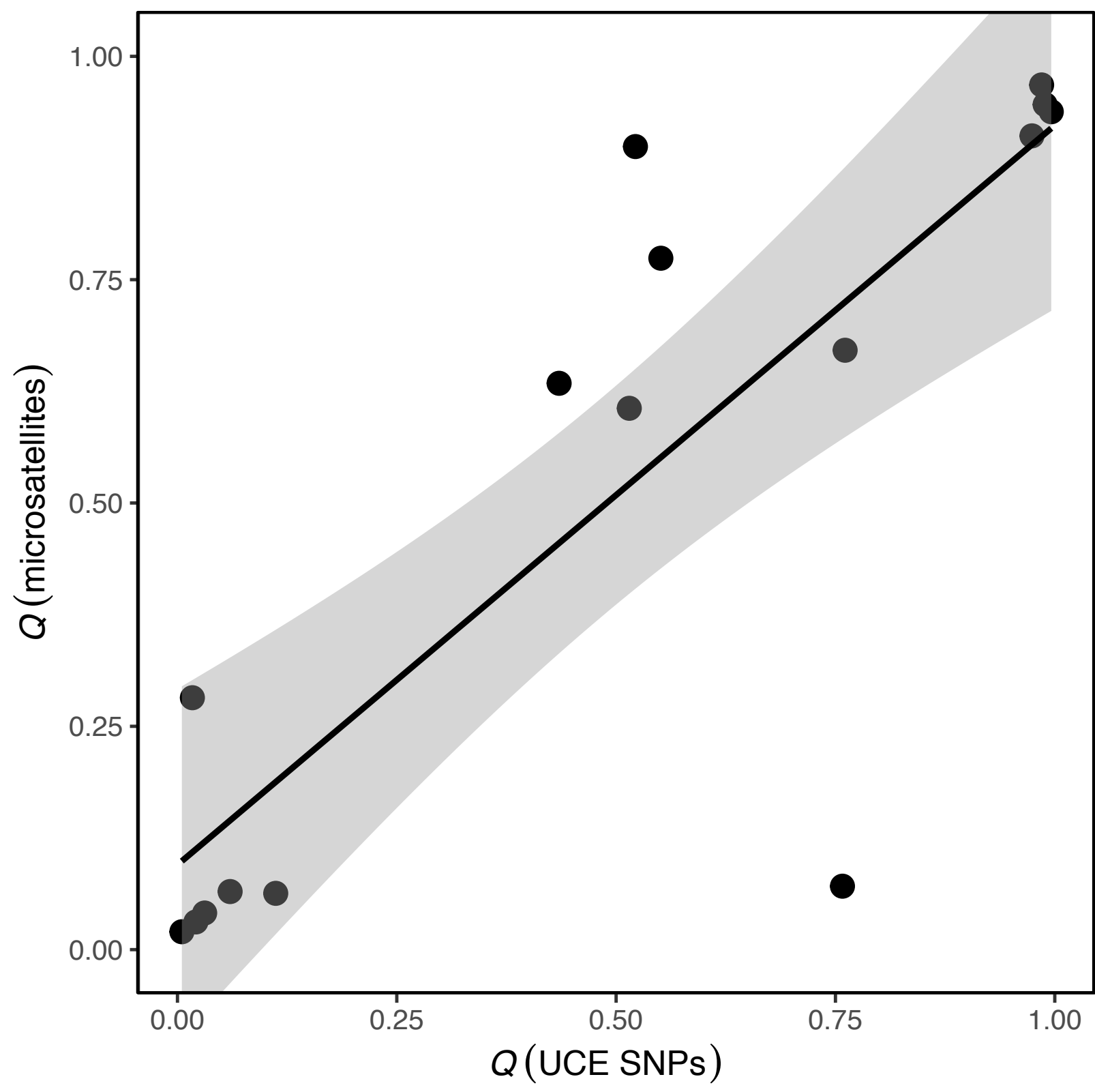

Supplement: Supplementary file 3 — Additional file 3: Figure S1. Linear regression of hybrid ancestry (Q scores) estimates. [file 13104_2019_4481_MOESM3_ESM.pdf]

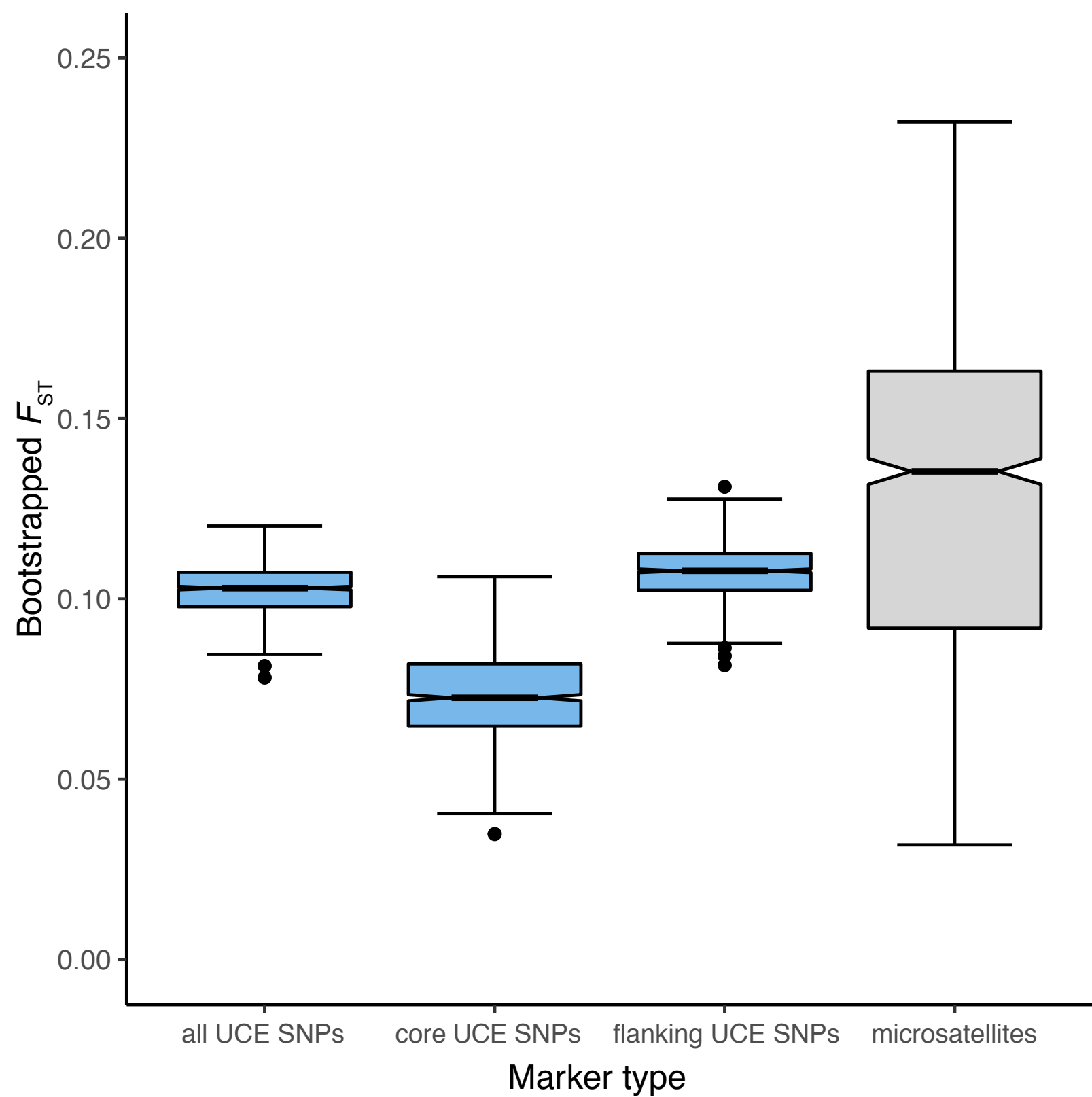

Supplement: Supplementary file 4 — Additional file 4: Figure S2. Bootstrapped average per-locus FST values. [file 13104_2019_4481_MOESM4_ESM.pdf]
