# Supplementary material for: Comparison of ultraconserved elements (UCEs) to microsatellite markers for the study of avian hybrid zones: a test in Aphelocoma jays
Source: BMC Res Notes. 2019 Jul 24;12:456. doi: 10.1186/s13104-019-4481-z (PMC6657088; doi:10.1186/s13104-019-4481-z)
Supplement: Supplementary file 5 — Additional file 5: Table S2. R2 values between hybrid index and phenotypic traits. [file 13104_2019_4481_MOESM5_ESM.pdf]

	Hybrid cline	Area of contact
Trait	Q_{SNPs}	Q_{SNPs}
Wing length	0.52*	ns
Tail length	ns	ns
Tarsus length	ns	ns
Bill length	ns	0.67*
Bill depth	ns	ns
Bill width	ns	ns
Morph PC1[1]	ns	ns
Morph PC2[2]	ns	ns
Morph PC3[3]	ns	ns

[1]PC1 describes general size.

[2]PC2 describes a trade-off between body and bill size.

[3]PC3 describes a trade-off between bill length and bill width.

ns = not significant, $*p < 0.05$.
